# Biography of Dr. T. B. Hamlin

**Published:** 1883-06

**Authors:** 


### Biographical.

### Biography of Dr. T. B. Hamlin.

Theodore B. Hamlin was born at Hamlin's Landing, since called Red Hook, on the Hudson river, in Dutchess County, New York, on June 24, 1810. His father was a small trader, not fixed permanently to any locality. Shortly after his birth the family removed to Windham, west of Catskill. Here the father soon died, leaving his wife and two sons in very destitute circumstances, Theodore being a little over two years old. Being without property or means of support, the widow and children went to her husband's father, at Sheffield, Massachusetts, where they remained between five and six years. They then moved to the house of the father's sister, Lucy Barnum, at Bainbridge, Chenango County, New York, where, after some years, his mother and only brother, Orin, died. We know nothing of how all these years were employed, nor by what influences Theodore was surrounded. We may safely conclude that his advantages were very few since, to use his own language, "at the age of sixteen years, he was without father, mother, brother or sister, money or education." Left thus alone in the world, and without material resources of any kind, he gave the first evidence of that decision and energy which afterwards characterized him by binding himself for three years to a watch repairer and silversmith named Marsh, in Oneida County, New York. He remained with Mr. Marsh two and a half years, and was treated with great kindness and consideration. Mr. Marsh sent him to school for six months, and at his desire, also procured instruction for him from the physician and acting dentist of the viliage. Thus early was his inclination turned towards the subsequent business of his life. Six months before the term of his service expired, on account of his rapid advancement and remarkable aptness for learning, Mr. Marsh released him his indenture, and procured for him a situation as journeyman in the largest jewelry and watch-making establishment in the city of Albany, and perhaps, at that time, in the United States. Mr. Terry, the proprietor, soon found he possessed unusual intelligence and skill, and shortly after his arrival and before he was nineteen years of age, made him foreman of the watch-making department. His thirst for knowledge, and continual close attention to his business soon began to show upon his health. He was never robust, small in stature, being about five feet seven inches high, he weighed but little over one hundred pounds, and inherited a frail constitution. He became dyspeptic and was compelled to seek relaxation. After spending a few months with an uncle at Red Hook, he partially recovered and returned to his work at Albany with Mr. Terry. The renewal of his labors brought on again his disease, and he was again compelled, and this time finally, to abandon his position.

From Albany he went to Lee, Massachusetts, and opened a small shop for watch repairing. Here at the age of twenty-two, he married the lady who now survives him. In addition to his regular work, and the study of dentistry, which he had never ceased, he commenced here the cultivation of bees, in which he afterwards became so proficient as to be an authority. The bleak climate of Massachusetts proved too rigorous for his constitution. He was attacked by rheumatism, and leaving his wife and daughter at Lynn, he spent a winter at New Orleans, working at his trade. During that winter, he invented what is still used and known as the centreing and pivoting tool in a watch-maker's lathe, by means of which, pivots can be replaced without making new work. Nothing has since been made to supercede the invention, and if a patent had been procured, it would have been productive of great profit. On the return of warm weather, Mr. Hamlin went back to his family at Lee, and at once determined to take up his residence in a milder climate. After some consideration, in the spring of 1835, being then nearly twenty-five years of age, he moved his family to Wytheville, Virginia, and commenced business as a practical jeweller, watch-maker and dentist. There was no one in the neighborhood qualified to perform dental operations, and as exigencies arose, Mr. Hamlin's skill was called into requsition. His ability soon became known, and his services in this direction being required more and more, the jewelry and watch making department gradually ceased to exist, and he devoted himself wholly to dentistry. His head-quarters being at Wytheville, he traveled in the counties of Smythe, Pulaski, Montgomery, Giles, Tazewell, Greenbrier and others, and regularly visited the watering places with which this region abounds. About this time the American Society of Dental Surgeons was organized at Baltimore. Mr. Hamlin soon after became a member and took an active part in the proceedings.

Such were his acquirements and his skill in the profession that his reputation had become firmly established, and he stood in the front rank. A few years later, the Baltimore College of Dental Surgery, the first of its kind in the world, was established at Baltimore, and he was among the first upon whom was conferred the honorary degree of D.D.S. He was instrumental in the organization of the Virginia Society of Dental Surgeons, the first chartered Dental Association in America, and having the power to confer the degree of Fellow of the Society. This organization was of great value in the State of Virginia, especially in restraining charlatanry and promoting union between the members of the profession.

Dr. Hamlin often reverted to these early Associations, as having been of great benefit to himself and to the profession. They gave character and dignity to Dental Surgery, and gave a powerful impetus to advancement both in the cultivation of the science and the practice of the art. While he resided at Wytheville, Dr. Hamlin purchased and improved a neat and comfortable place of residence, besides other property, and employed his leisure time in the ardent and enthusiastic study and cultivation of bees. Here also were born to him three children, besides the the daughter, born at Lee. After residing ten years at Wytheville, in 1845 he removed to Tuscumbia, Alabama, and in 1847, to Nashville, Tennessee. At Nashville he at once took a leading position. He purchased eligible and valuable property in a central location, which had been built for dental purposes, and from the beginning had a lucrative practice. He continued to practice alone until the year 1849 when he formed a partnership with W. H. Morgan, D.D.S., before that time of Russellville Kentucky, with whom he remained until he retired from practice. The partners were peculiarly adapted to each other. Both had lacked early training—had pushed forward into the profession, in spite of all obstacles, and had acquired skill and information by the most diligent and unremitted labor. Apart from their earnestness, skill and diligence, they were as diverse as possible. Dr. Hamlin, small, weak, frail and silent. Dr. Morgan, large, powerful, vigorous, pleased and pleasing in society, and conversationally eloquent. They could not but be harmonious and successful. In his latter days Dr. Hamlin often spoke of his association with Dr. Morgan as the most pleasant period of his professional life, and until his death Dr. Morgan continued to be his firmest and most valued friend. In notes on his life, written at his dictation, he says: "We labored together more than ten years, both pleasantly and profitably."

For some years previous to 1860, Dr. Hamlin's health had been failing. The dyspepsia acquired in his youth at the work bench in Albany had never been eradicated, and at intervals gave undoubted evidence of its presence. He had also been afflicted with what was supposed to be "Bright's disease," and had spent a summer at Newport, with hope of relief. In 1860, it became certain that he could not longer practice his profession and hope to restore his health, he therefore disposed of the business to Dr. Morgan, dissolved the partnership, and retired to his residence in Edgefield, across the river from Nashville. With the exception of a few months in 1864 or 5, when he made merely an experiment to find out his own powers, this closed Dr. Hamlin's career as a dentist. During the war he resided in Edgefield, making frequent Northern tours in quest of health. In 1866, he purchased a large and valuable farm, nine miles north of Nashville, at Edgfield Junction, and devoted his entire attention to his apiary and the propagation of fruit trees.

As a keeper of bees, Dr. Hamlin was remarkably successful. The care and safety with which he manipulated them was astonishing, and was only surpassed by the extent and accuracy of his knowledge of their habits. In 1867, he commenced the importation of queen bees from Italy and Germany, and from them propagated and distributed large numbers. At the time of his death his apiary contained several hundred hives.

Dr. Hamlin was, at different times, Worshipful Master of three Masonic Lodges, and for more than twenty years a Knight Templar, and member of Nashville Commandery. He was a decided and sincere Christian, and early in life connected himself with the Presbyterian Church, of which he was a member at the time of his death, on May 24th, 1874.

As an author, Dr. Hamlin did not much appear. In 1848 he published a small, but valuable treatise, entitled "Importance and Care of the Teeth," and afterwards another, called "Quackery Unmasked." In 1871 he also published a small work on "Improved Bee Culture," which is regarded as valuable in that department.

The life of Dr. Hamlin is especially noticeable from the fact that by earnest, persistent effort and determination, he overcame difficulties, at any one of which an ordinary man would have been appalled. Poverty, ignorance, disease, all combined to draw him from his purpose, but he triumphed over all, and not only maintained himself, but rose superior to most of those with whom he was associated.

In all the relations of life, he was a true man. As a dentist he was a skillful operator, possessing great native ingenuity and untiring industry. His integrity and strict justice in whatever he engaged, always ensured to him profound respect, and his energy and force contributed materially to the advancement of anything in which he was interested.

His last days were quiet, calm and undisturbed by bodly pain or vain regrets. He had lived an active, busy life—had improved his opportunity to the best of his ability, and had little cause to reproach himself. More truly than almost any one within our knowledge, he could say that he had done his might in every position in which he had been placed, and had "finished his course." He was buried in Mount Olivet Cemetery, at Nashville, with Masonic and Templar honors, on May 25th, 1874.

"He has lived his life, and that which he has done,
May He within himself make pure."

